# Dynorphin and κ-Opioid Receptor Dysregulation in the Dopaminergic Reward System of Human Alcoholics

**DOI:** 10.1007/s12035-017-0844-4

**Published:** 2018-01-30

**Authors:** Igor Bazov, Daniil Sarkisyan, Olga Kononenko, Hiroyuki Watanabe, Tatiana Yakovleva, Anita C. Hansson, Wolfgang H. Sommer, Rainer Spanagel, Georgy Bakalkin

**Affiliations:** 1 Division of Biological Research on Drug Dependence, Department of Pharmaceutical Biosciences, Uppsala University, Box 591, BMC Husargatan 3, SE-75124 Uppsala, Sweden; 2 Institute of Psychopharmacology, Central Institute of Mental Health, Medical Faculty Mannheim, Heidelberg University, 68159 Mannheim, Germany

**Keywords:** Alcohol addiction, Post-mortem human brain tissue, Nucleus accumbens, Reward system, Dysphoria, Co-expression of gene clusters, κ-opioid receptor, Dynorphin, D1-pathway, D2-pathway

## Abstract

Molecular changes induced by excessive alcohol consumption may underlie formation of dysphoric state during acute and protracted alcohol withdrawal which leads to craving and relapse. A main molecular addiction hypothesis is that the upregulation of the dynorphin (DYN)/κ-opioid receptor (KOR) system in the nucleus accumbens (NAc) of alcohol-dependent individuals causes the imbalance in activity of D1- and D2 dopamine receptor (DR) expressing neural circuits that results in dysphoria. We here analyzed post-mortem NAc samples of human alcoholics to assess changes in prodynorphin (*PDYN*) and KOR (*OPRK1*) gene expression and co-expression (transcriptionally coordinated) patterns. To address alterations in D1- and D2-receptor circuits, we studied the regulatory interactions between these pathways and the DYN/KOR system. No significant differences in *PDYN* and *OPRK1* gene expression levels between alcoholics and controls were evident. However, *PDYN* and *OPRK1* showed transcriptionally coordinated pattern that was significantly different between alcoholics and controls. A downregulation of *DRD1* but not *DRD2* expression was seen in alcoholics. Expression of *DRD1* and *DRD2* strongly correlated with that of *PDYN* and *OPRK1* suggesting high levels of transcriptional coordination between these gene clusters. The differences in expression and co-expression patterns were not due to the decline in neuronal proportion in alcoholic brain and thereby represent transcriptional phenomena. Dysregulation of DYN/KOR system and dopamine signaling through both alterations in co-expression patterns of opioid genes and decreased *DRD1* gene expression may contribute to imbalance in the activity of D1- and D2-containing pathways which may lead to the negative affective state in human alcoholics.

**Electronic supplementary material:**

The online version of this article (10.1007/s12035-017-0844-4) contains supplementary material, which is available to authorized users.

## Introduction

Worldwide, more than 2 billion people consume alcohol and around 6% of adults have an alcohol-use disorder [1]. Alcohol addiction is defined as a chronically relapsing disorder characterized by compulsive craving that derives from dysregulation of reward functions and from impaired top-down control due to neurotoxic effects of alcohol intoxication and withdrawal in the prefrontal cortex and related brain regions following excessive alcohol consumption [2–4]. Especially during withdrawal, a negative emotional state—characterized by dysphoria and distress—occurs which is seen as a major driver for craving and relapse [3]. At least two molecular mechanisms may contribute to a state of dysphoria and distress. One mechanism involves a pathological engagement of extra-hypothalamic corticotropin-releasing hormone (CRH) transmission and CRH receptor 1 signaling [5] whereas a second mechanism involves an upregulation of the dynorphin (DYN) and κ-opioid receptor (KOR) system in the nucleus accumbens (NAc) [6–9].

Four key discoveries show that the accumbal DYN/KOR system is critical for mediating a state of dysphoria. First, the application of a KOR agonist produces a strong place aversion in laboratory animals [10]. This finding is interpreted in a way that activation of the KOR produces an aversive state. Second, the ingestion of a KOR agonist by human volunteers produces a very pronounced state of dysphoria and elicits psychotomimetic effects [11]. Third, KOR-induced aversion (dysphoria) is explained on the molecular level by the finding that the dopaminergic reward system is directly regulated by DYN and KOR. Thus, activation of KORs onto dopaminergic terminals within NAc leads to a strong decrease in dopamine (DA) levels [12, 13] and a hallmark of acute alcohol withdrawal is strongly reduced DA levels ([14], but see also [15]). Finally, a recent study identified the DYN microcircuitry within NAc that drives aversion [16]. Within NAc, dopamine and glutamate neurons impinge onto D1 dopamine receptor-containing medium spiny neurons (DRD1-MSNs) and onto DRD2-MSNs. There seems to be a dichotomy in a way of a more predominate role for DRD1-MSNs in producing reinforcement whereas activation of DRD2-MSN in producing aversion [17–22]. The proposed microcircuitry suggests that presynaptic inhibition by KORs of inhibitory synapses on DRD2-MSNs enhances integration of excitatory drive. This leads to a disinhibition of DRD2-MSNs and thereby favors this pathway which then drives aversion [23].

Preclinical studies led to the hypothesis that activation and upregulation of the accumbal DYN/KOR system following chronic alcohol consumption may be a basis for the negative motivational effects (i.e., dysphoria and distress) observed during alcohol withdrawal [6, 7, 24]. In particular, in alcohol-dependent animals, a hyperactive DYN/KOR system may mediate the negative motivational effects during withdrawal [8, 25–27]. In line with this hypothesis is that excessive, compulsive-like alcohol intake can be blocked by KOR antagonists through their effects in NAc [26–29]. Currently, we have no knowledge about the status of the DYN/KOR system in NAc in the alcohol-addicted human brain. The aim of the present study was to examine whether the DYN/KOR system undergoes adaptive changes in NAc of human alcoholics. Along with average expression levels, co-expression patterns of the prodynorphin (*PDYN*) and KOR (*OPRK1*) genes were compared between alcoholics and controls. To address alterations in the proposed microcircuitry within NAc which seem to be critically involved in mediating a dysphoric state [23], we further studied the regulatory interactions between the DYN/KOR and DRD1/DRD2 systems in the human NAc of alcoholics and controls.

A number of neurons in NAc are markedly reduced in alcoholics as shown by analyses of neuronal proportion quantified from DNA methylation profiles and expression of a neuronal marker [4]. To attribute potential expression changes to either transcriptional events or changes in cell composition in alcoholics, we analyzed the effects of the decline in neuronal proportion on messenger RNA (mRNA) alterations in alcoholic brain.

## Materials and Methods

### Human Samples

Human brain tissue samples were collected at the New South Wales Brain Tissue Resource Centre, University of Sydney, Australia (http://sydney.edu.au/medicine/pathology/btrc/; see Table 1 for summary and Table S1 for detailed information). Tissue samples from 42 DSM-IV alcoholic and 50 control subjects, all males of European descent, were analyzed. Alcoholics were the subjects that met Diagnostic and Statistical Manual for Mental Disorders, 4th edition (DSM-IV) criteria for alcohol abuse or alcohol dependence and consumed 226 ± 24 g (mean ± S.E.M.) of ethanol per day in average for the majority of their adult lives [30]. Controls had either abstained from alcohol completely or were social drinkers who consumed 16 ± 3 g of ethanol per day on average. Methods used to classify alcoholics were described previously [30, 31]. Cases with a prolonged agonal life support and a history of cerebral infarction, head injury, or neurodegenerative disease (e.g., Alzheimer’s disease) were excluded. Each sample of NAc included its lateral, medial, and central parts and was dissected from the region of the caudate-putamen junction located inferior to the internal capsule and anterior to the anterior commissure. Alcohol was detected in blood of 24 subjects; average for the group blood alcohol concentration (BAC) was 0.16 ± 0.14 g/100 mL at the time of death (the “intoxicated” group). The “not-intoxicated” group consisted of 43 subjects with BAC levels below the detection limit and 25 subjects for whom information was absent. Information on smoking status was available for 95% of subjects (Table S1; “ex-smokers” were grouped with “non-smokers”). Informed written consent for autopsy was obtained from the next-of-kin, and collection was approved by the Human Research Ethics Committees of the Sydney Local Health District (X15-0199) and the University of Sydney. The study was approved by the Swedish Central Ethical Review Board.

**Table 1:** Summary of demographic data and tissue characteristics of human subjects (for details, see Supplementary Table S1)

Cohort	Number	Age	PMI	pH	RQI	Neuronal proportion^a^
Controls	50	53.8 ± 9.0	30.3 ± 12.5	6.6 ± 0.3	8.2 ± 0.9	0.25 ± 0.04
Alcoholics	42	56.1 ± 8.4	37.6 ± 15.0	6.5 ± 0.3	7.4 ± 1.3	0.22 ± 0.07
*P* value		n.s.	0.013	n.s.	0.002	0.014

Values are means ± SD *SD* standard deviation. Unpaired *t* test was used to calculate *P* values

*SD* standard deviation, *N* number of subjects, *Age* age in years, *PMI* post-mortem interval in hours, *pH* brain pH, *RQI* RNA quality indicator, *n.s.* not significant

^a^Data are taken from our previous paper [4]

### RNA Purification

Total RNA was purified using RNeasy Lipid Tissue Mini Kit (Qiagen) and treated with RNase-free DNase I (Qiagen) on-column according to the manufacturer’s recommendations. RNA concentrations and 260/280 and 260/230 ratios were measured with a Nanodrop. RNA Quality Indicator (RQI) was measured using Bio-Rad Experion (Bio-Rad Laboratories) with Eukaryote Total RNA StdSens assay according to the manufacturer’s protocol. Five hundred nanograms of RNA was reverse-transcribed to complementary DNA (cDNA) in duplicates with the High Capacity RNA-to-cDNA kit (Applied Biosystems) according to the manufacturer’s recommendations.

### Gene Expression Analysis

TaqMan assays (Applied Biosystems) for *DRD1* (Hs00265245_s1), *DRD2* (Hs00241436_m1), *OPRK1* (Hs00175127_m1), *PDYN* (Hs00225770_m1), *POLR2A* (Hs00172187_m1) and *RPLP0* (Hs99999902_m1) were used. cDNAs were mixed with TaqMan assay and iTaq Universal Probes supermix (Applied Biosystems) for qPCR with a CFX96 Real-Time Detection System (Bio-Rad) according to the manufacturer’s instructions. mRNA levels of gene of interest were normalized to geometric mean of expression levels of two control genes *POLR2A* and *RPLP0* selected by geNORM program (https://genorm.cmgg.be/) ([32]; see also our studies [33–35]). In each experiment, internal control gene stability measure M [32] was controlled for and did not exceed the limit of 0.5.

### DNA Methylation Analysis

Total tissue DNA was purified from human brain samples using the DNeasy Blood & Tissue kit (Qiagen) and bisulfite converted using the EZ DNA methylation Gold kit (Zymo Research) according to the manufacturer’s instructions. Methylation profiling was performed using the Illumina Infinium HumanMethylation450 BeadChip assay (450 K) by the SNP&SEQ Technology Platform at Science for Life Laboratory (Sweden). Data was analyzed using the *R*/*Bioconductor* 3.3 package *minfi* 1.18.2 [36]. Poor-quality samples (mean detection *P* value > 0.01) were discarded; probes with a detection *P* value > 0.01 in at least one sample within a dataset were also discarded, as were X and Y chromosome probes, probes with SNPs at the CpG or single-base extension site, and the cross-reactive probes identified by [37].

### Computation of Neuronal Proportions

Genome-wide DNA methylation data for 482,421 CpGs in total tissue DNA, profiled with 450 K assay, was processed using R package Cell EpigenoType Specific *CETS* (3.0.3) package [38]. *CETS* predicts neuronal proportions from methylation levels of top 10,000 marker CpGs that demonstrated the most significant methylation differences between neuronal and non-neuronal DNA. In the original study [38] devoted to the development of *CETS*, these CpGs have been identified by genome-wide analysis of DNA prepared from neuronal and non-neuronal nuclei isolated by fluorescence-activated nuclear sorting (FANS) using antibodies against neuronal nuclear antigen NeuN transcribed from the *RBFOX3* gene.

### Statistical Analysis

**Fig. 1:**
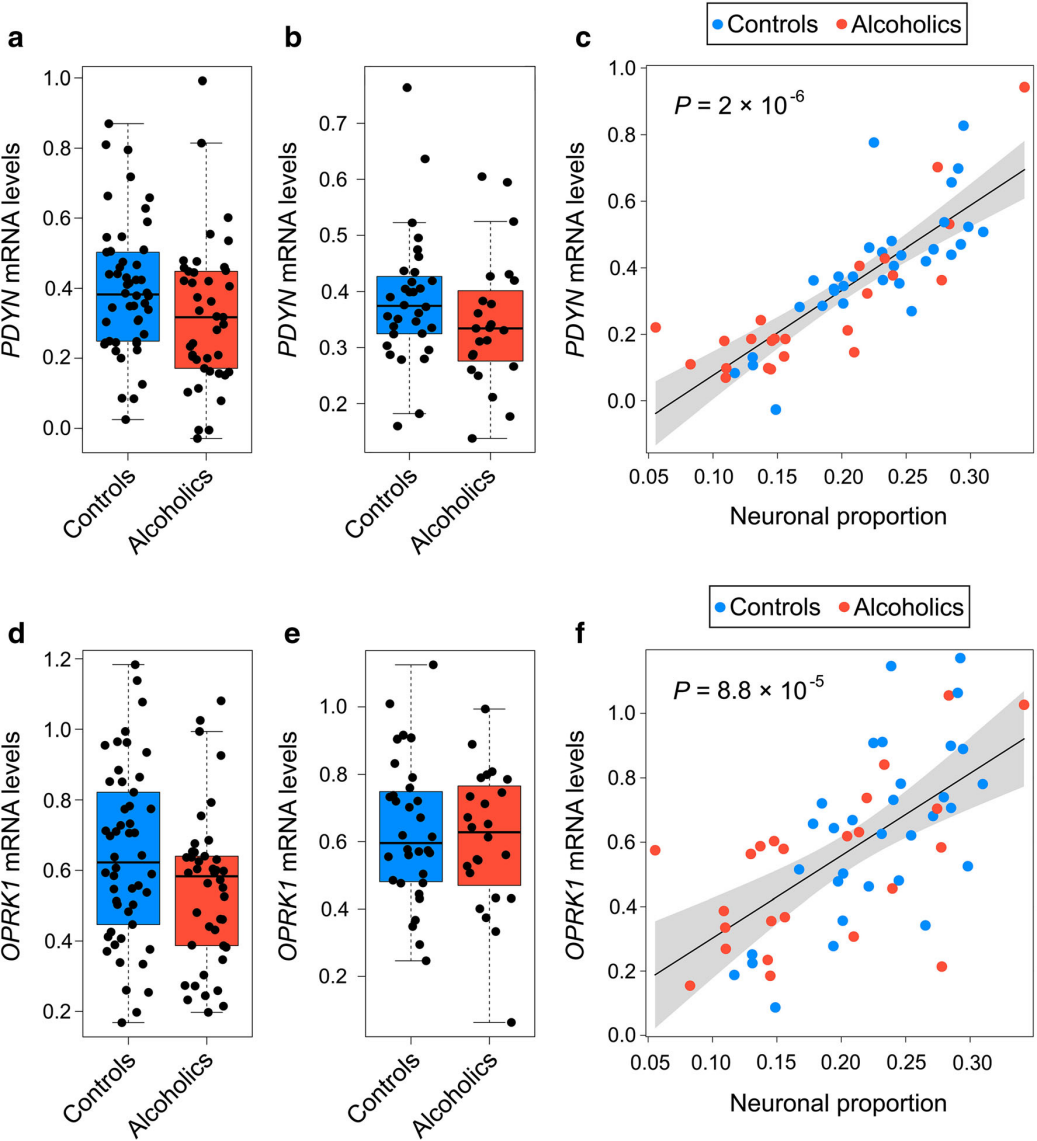
Effects of alcoholism on *PDYN* and *OPRK1* expression levels in NAc. **a**, **d** Presented are data on whole tissue *PDYN* and *OPRK1* mRNA levels for 50 controls and 42 alcoholics. **b**, **e** Presented are data on whole tissue *PDYN* and *OPRK1* mRNA levels adjusted for changes in neuronal proportion for 32 controls and 24 alcoholics. **c**, **f** Correlations between neuronal proportion and *PDYN* (**c**) or *OPRK1* (**f**) mRNAs. Statistical analysis was performed by one-way ANCOVA; *P* values were calculated by ordinary bootstrap with 5 × 10^5^ non-parametric resampling of cases. mRNA levels are shown in arbitrary units. In boxplots, the middle line is the median, box spans the interquartile range (IQR), and whiskers extend 1.5 × IQR from box limits. Lines and shading represent the estimated slopes and 95% confidence intervals (CIs), respectively

**Fig. 2:**
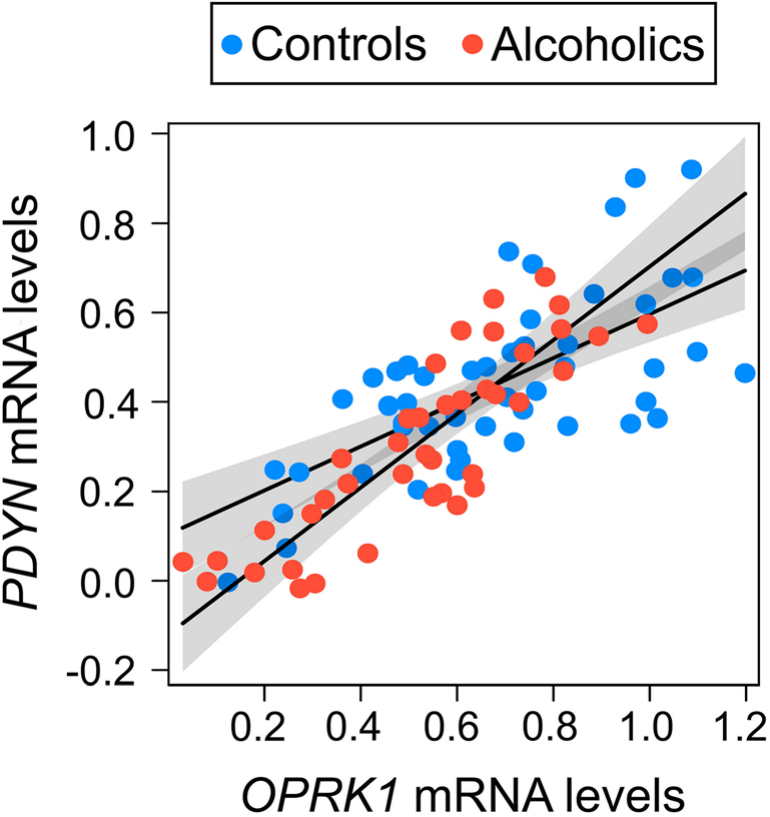
Relationship between *PDYN* and *OPRK1* in alcoholics (*N* = 42) and controls (*N* = 50). The slope of the regression line was steeper in alcoholics than in controls (two-way ANCOVA; *P* values were calculated by ordinary bootstrap with 5 × 10^5^ non-parametric resampling of cases; *P* = 0.011). mRNA levels are shown in arbitrary units. Lines and shading denote alcoholism slope estimates and 95% CI, respectively

**Fig. 3:**
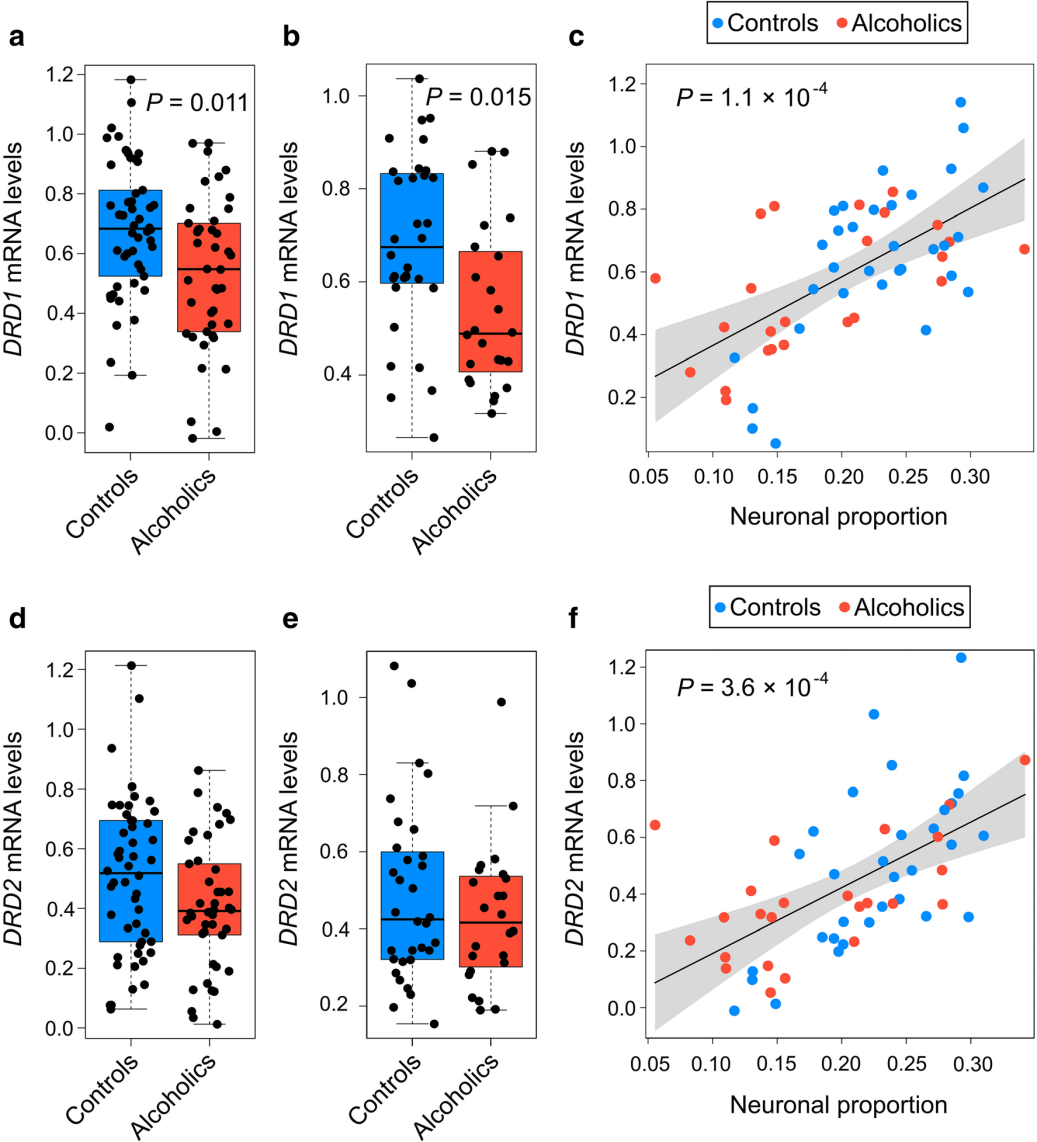
Effects of alcoholism on *DRD1* and *DRD2* expression levels in NAc. **a**, **d** Presented are data on whole-tissue *DRD1* and *DRD2* mRNA levels for 50 controls and 42 alcoholics. **b**, **e** Presented are data on whole-tissue *DRD1* and *DRD2* mRNA levels adjusted for changes in neuronal proportion for 32 controls and 24 alcoholics. **c**, **f** Correlation between neuronal proportion and *DRD1* mRNA (**c**) or *DRD2* mRNA (**f**). Statistical analysis was performed by one-way ANCOVA; *P* values were calculated by ordinary bootstrap with 5 × 10^5^ non-parametric resampling of cases. mRNA levels are shown in arbitrary units. In boxplots, middle line is the median, box spans the interquartile range (IQR), and *whiskers* extend 1.5 × IQR from box limits. Lines and shading represent the estimated slopes and 95% CI, respectively

**Fig. 4:**
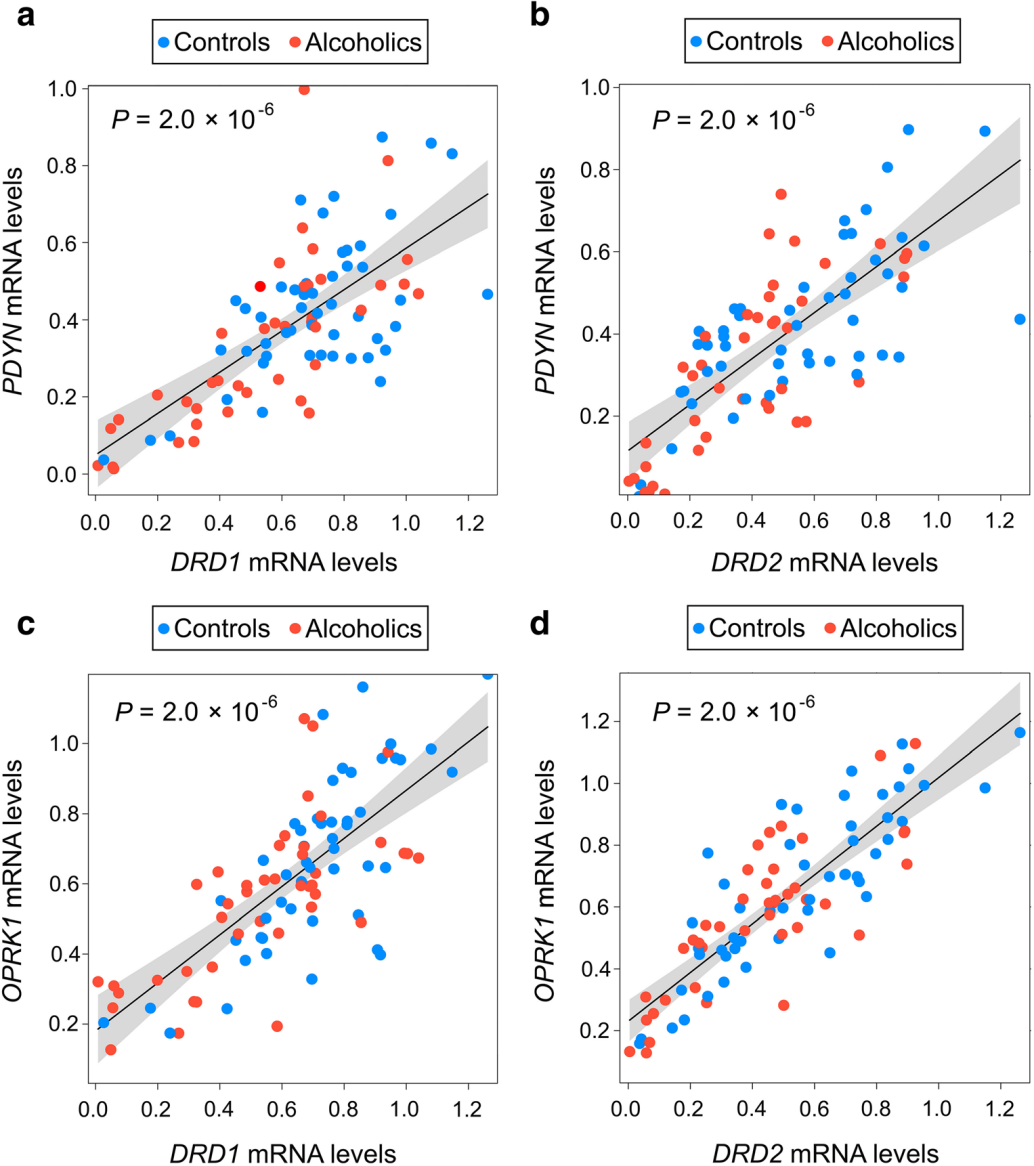
Correlation between expression of the opioid *PDYN* and *OPRK1* genes and the dopamine receptor *DRD1* and *DRD2* genes in NAc. **a**–**d** Relationship between *PDYN* and *DRD1* (**a**), *PDYN* and *DRD2* (**b**), *OPRK1* and *DRD1* (**c**), and *OPRK1* and *DRD2* (**d**) in combined sample of controls and alcoholics (*N* = 92 subjects). Statistical analysis was performed by one-way ANCOVA; *P* values were calculated by ordinary bootstrap with 5 × 10^5^ non-parametric resampling of cases. mRNA levels are shown in arbitrary units. Lines and shading represent alcoholism slope estimates and 95% CI, respectively

Statistical analysis was performed using R version 3.3.2 (http://www.R-project.org/). For analysis of gene expression, linear regression models were constructed adjusting for age, PMI, brain pH, RQI, and alcoholism (Figs. 1a, d and 3a, d). Gene expression was then further adjusted for neuronal proportion (Figs. 1b–f and 3b–f). Two-way ANCOVA was performed to analyze effects of interaction between alcoholism and *OPRK1* (KOR) mRNA levels on *PDYN* mRNA levels while also adjusting for age, PMI, brain pH, and RQI (Fig. 2). Analysis of correlations between each *PDYN* and *OPRK1* (KOR) and *DRD1* and *DRD2* genes included adjustment for age, PMI, brain pH, and RQI, and also for another *DRD* gene (Fig. 4). Significance of differences was further controlled by adjusting for smoking status (either smoker or non-smoker), the presence of alcohol (either positive or negative BAC), alcohol withdrawal signs (yes or no), DSM-V severity of alcohol-use disorder (mild, moderate or severe), and average daily and lifetime alcohol consumption. Overly influential points with Cook’s distance ≥ 1.0 were removed from the analysis of the above models [39]. R package *effects* was used to construct effect displays (component and residual plots). Bootstrapped *P* values and bias-corrected and accelerated bootstrap percentile 95% confidence intervals (CIs) for regression coefficients, both of which do not require the assumption of normality, were estimated using *car* package with *R* = 5 × 10^5^ resampled cases [40]. A significance level of *P* < 0.05 was accepted as statistically significant, and all tests were two-tailed.

## Results

Forty-two alcoholic and 50 control subjects were analyzed in the study. Effects of alcoholism on the whole tissue levels of *PDYN*, *OPRK1*, *DRD1*, and *DRD2* mRNAs in NAc were examined after adjusting for demographical data and tissue characteristics including age, PMI, brain pH, and RQI. Age and brain pH did not differ between the groups, while PMI and RQI differed significantly (Table 1). Tissue expression levels may differ due to changes in cell composition if genes of interest are transcribed in specific cell types. Moreover, we recently demonstrated that the number of neurons in NAc is markedly reduced in alcoholics [4]. Therefore, neuronal proportion was computed by using genome-wide DNA methylation data and was included as confounding factor in the analysis (for details, see [4] and Table 1).

### No Effects of Alcoholism on *PDYN* and *OPRK1* mRNA Expression

We first examined whether expression of *PDYN* and *OPRK1*—calculated for the whole tissue and also adjusted for changes in cell composition—was affected by alcoholism. No significant differences between alcoholics and controls were evident in the tissue levels of *PDYN* (*P* = 0.134) or *OPRK1* mRNA (*P* = 0.097) (Fig. 1a, d). The results did not change when these levels were adjusted for changes in neuronal proportion (*PDYN*, *P* = 0.398; *OPRK1*, *P* = 0.689) (Fig. 1b, e). As expected from our previous study [4], alcoholics had in general lower neuronal proportions than did the controls and both *PDYN* and *OPRK1* mRNAs strongly and significantly correlated with neuronal proportion (ANCOVA estimated by bootstrap resampling for *PDYN*, *P* = 2 × 10^−6^ and for *OPRK1*, *P* = 8.8 × 10^−5^) (Fig. 1c, f). Effects of alcoholism on gene expression were not significant when BAC (*PDYN*, *P* = 0.062; *OPRK1*, *P* = 0.068; no difference between intoxicated and non-intoxicated subjects) was included as covariate. No significant effects on gene expression were found for alcohol withdrawal signs (*PDYN*, *P* = 0.317; *OPRK1*, *P* = 0.643), severity of alcohol abuse (*PDYN*, *P* = 0.442; *OPRK1*), average daily consumption (*PDYN*, *P* = 0.383; *OPRK1*, *P* = 0.661), or lifetime consumption (*PDYN*, *P* = 0.703; *OPRK1*, *P* = 0.910).

### Altered Correlation of *PDYN* and *OPRK1* Expression in Alcoholics

The balance between concentrations of a receptor and its ligand may be established already at the transcriptional level [41]. The *PDYN* and *OPRK1* genes may be co-expressed (i.e., transcriptionally co-regulated) in spite of only partial overlap of their anatomical expression patterns—D1-MSNs transcribe both genes, while D2-MSNs only *OPRK1* [23, 42–44]). Their co-expression pattern may be affected upon transition from normal to a pathological, alcoholic state. To address this hypothesis, we compared the slopes of the regression lines for *PDYN* and *OPRK1* between controls and alcoholics.

*PDYN* mRNA significantly correlated with *OPRK1* mRNA (*P* = 2 × 10^−6^), and there was a significant effect of interaction between alcoholism and *PDYN*–*OPRK1* correlation (*P* = 0.011; Fig. 2). Significant effect of interaction between alcoholism and *PDYN*–*OPRK1* correlation (*P* = 0.018) was found when BAC levels were included as covariates. In other words, correlation between *PDYN* and *OPRK1* expression significantly differed between alcoholics irrespective to intoxication status and controls, and the slope of regression line was steeper in alcoholics than in controls (Fig. 2), suggesting that alcoholics with high *OPRK1* expression have greater *PDYN* mRNA levels than respective controls. Because the activation of a neuropeptide receptor depends on the concentration of its peptide ligand, which may be produced in shortage or in excess relative to the receptor level [41], these results suggest that alcoholics with high *PDYN* and *OPRK1* expression levels are characterized by a stronger KOR activation due to enhanced DYN levels compared to controls.

### A Decline in *DRD1* but not *DRD2* and Tightly Coordinated Co-Expression of *PDYN* and *OPRK1* with *DRD* Genes may Mediate a Dysphoric State

To address alterations in the proposed microcircuitry within NAc which seem to be critically involved in mediating a dysphoric state [23], we further studied the regulatory interactions between the DYN/KOR and DRD1/DRD2 systems in the human NAc of alcoholics and controls. Thus, we examined whether transcription of the opioid *PDYN* and *OPRK1* genes and dopamine receptor genes is coordinated in this neural microcircuitry, and if so, whether their co-expression patterns are affected by alcoholism.

First, we examined whether *DRD1* and *DRD2* expression is affected by alcoholism, and whether alcoholism effects may be due to neuronal proportion in the alcoholic brain. The *DRD1* mRNA tissue levels were significantly 1.26-fold lower in alcoholics compared to controls (*P* = 0.011; Fig. 3a); adjustment for changes in cell composition did not affect the significance difference between alcoholics and controls (*P* = 0.015; Fig. 3b). Effect of alcoholism on *DRD1* expression was virtually the same when BAC (1.28-fold, *P* = 0.023; no difference between intoxicated and non-intoxicated subjects) or smoking status (1.3-fold, *P* = 0.009; no difference between smokers and non-smokers) were included as covariates. In contrast to *DRD1*, no significant effects of alcoholism on *DRD2* mRNA levels were found (*P* = 0.087; corrected for neuronal proportion, *P* = 0.425) (Fig. 3d, e). No significant effects on expression levels were found for alcohol withdrawal signs (*DRD1*, *P* = 0.824; *DRD2*, *P* = 0.273), severity of alcohol abuse (DRD2, *P* = 0.319), average daily consumption (*DRD1*, *P* = 0.935; *DRD2*, *P* = 0.893), or lifetime consumption (*DRD1*, *P* = 0.674; *DRD2*, *P* = 0.992). A significant effect of severity of alcohol abuse on *DRD1* expression (*P* = 0.024) was not confirmed by post hoc tests (the most significant *P* = 0.053 between controls and alcoholics with severe disorder).

Both *DRD1* and *DRD2* mRNA significantly correlated with neuronal proportion (*DRD1*, *P* = 1.1 × 10^−4^; *DRD2*, *P* = 3.6 × 10^−4^) (Fig. 3c, f). Thus, in NAc of alcoholics, the *DRD1* but not *DRD2* mRNA levels were lower, and this effect was not due to the changes in cell composition, smoking status, alcohol intoxication, or other clinical variables.

Finally, correlations between *DRD1*–*PDYN* (*P* = 2 × 10^−6^), *DRD2*–*PDYN* (*P* = 2 × 10^−6^), *DRD1*–*OPRK1* (*P* = 2 × 10^−6^), and *DRD2*–*OPRK1* (*P* = 2 × 10^−6^) were positive and highly significant (Fig. 4a–d). No effects of alcoholism × *DRD1* or alcoholism × *DRD2* interactions on these correlations were revealed suggesting that alcoholics and controls did not differ in strength and slope of these correlations. Thus, these four genes demonstrated robust co-expression patterns in spite of *PDYN* and *DRD2* expression in different neuronal subtypes.

## Discussion

The dominant molecular concept of addiction postulates that DYN is upregulated in NAc by chronic intake of addictive substances such as alcohol and that these molecular changes contribute to escalated drug and alcohol consumption [45–48]. Studies including optogenetic and electrophysiological analyses demonstrated a role for KOR in NAc in mediating a negative affective state along with enhanced stress reactivity and deciphered the underlying pathway- and cell type-specific mechanisms [15, 16, 23]. In particular, it has been proposed that presynaptic inhibition by KORs of inhibitory synapses on DRD2-MSNs enhances integration of excitatory drive and thereby leads to a disinhibition of DRD2-MSNs which then results in an aversive response [23]. However, whether the DYN/KOR system undergoes adaptive changes upon transition to alcohol addiction in human NAc is not known. It is also not known whether an imbalance in D1/D2-MSNs activity following KOR activation observed in laboratory rodents translates to the human brain.

Animal studies of *Pdyn* mRNA and/or peptides in NAc after “binge” and continuous exposure to cocaine, morphine, heroin, amphetamine, and alcohol were inconclusive. No changes [49–55], upregulation [56–62], or downregulation at early time point after treatment [63, 64] were reported. The *Oprk1* mRNA levels were reduced in the rodent NAc in response to repeated ethanol and/or cocaine exposure [65] or showed no changes in NAc of animals repeatedly treated with heroin [51].

Post-mortem molecular human studies revealed *PDYN* downregulation in NAc in heroin addicts [66] or no changes in *PDYN* or dynorphin and KOR protein expression in NAc of cocaine addicts [67, 68], while the *PDYN* mRNA levels were decreased in dorsal striatum in alcoholics [35] and in caudate nucleus of cocaine addicts carrying *PDYN* SNP variant associated with cocaine/alcohol co-dependence [69]. Here, we now show that human alcoholics do not exhibit alterations in *PDYN* and *OPRK1* mRNA levels in NAc compared to controls; this finding remains unchanged if neuronal proportion and several other confounding factors such as age, PMI, brain pH, and RQI were controlled for. However, we found that *PDYN–OPRK1* co-expression pattern within NAc is significantly altered in alcoholics (Fig. 2). The effect of alcoholism on the slope of the correlation between *PDYN* and *OPRK1*, manifested as significant alcoholism × *OPRK1* interaction, suggests that alcoholics with high *OPRK1* expression have greater *PDYN* mRNA levels than respective controls. Given that activation of KORs depends on the concentration of their endogenous DYN ligand [41], these results suggest a stronger KOR activation due to enhanced DYN levels in alcoholics with high *PDYN* and *OPRK1* expression levels compared to controls.

Tejeda et al. [23] provide a microcircuitry-based framework for NAc wherein DYN-mediated modulation of excitatory and inhibitory synapses differentially alters activity of D1- and D2-MSNs. Glutamatergic synapses on D1-MSNs but not on D2-MSNs are sensitive to KOR inhibition. Within this local microcircuit, MSN collaterals are controlled by DYN via presynaptic KOR-mediated inhibition of GABA release. DYN inhibits D1-MSN collaterals stronger than D2-MSN collaterals. The overall outcome of DYN actions is inhibition of synaptic drive of D1-MSNs and disinhibition of the excitatory drives of D2-MSNs that underlies a shift to an aversive state [23].

**Fig. 5:**
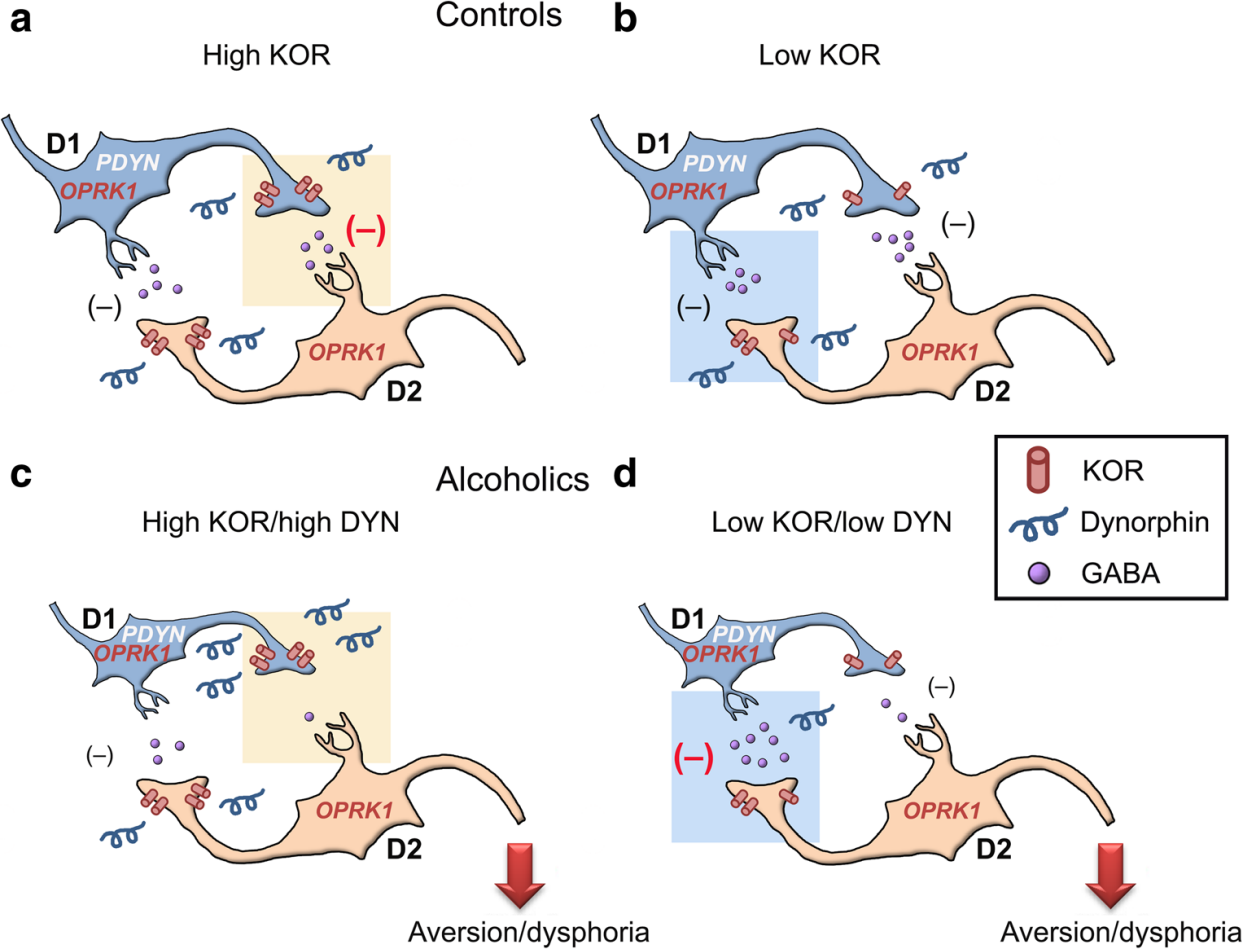
Functional implications of transcriptional changes in expression and co-expression patterns of the opioid *PDYN* and *OPRK1* genes and the dopamine receptor *DRD1* and *DRD2* genes, for the shift to aversive and dysphoric state in alcoholics. Downregulation of *DRD1* expression and binding potential (present study and [15]) in alcoholics may reflect the decline in DRD1-mediated synaptic activity or the number of D1-MSNs, and that in turn may lead to disinhibition of D2-MSNs, in which activation results in dysphoria and aversive behavior. Dynorphins regulate information processing in the MSN local microcircuitry by inhibiting GABA release from D1- and D2-MSN collaterals [23]. Changes in the *PDYN*/*OPRK1* co-expression pattern suggest that in subgroup of alcoholics with high DYN and KOR expression, the D2-MSN output may be preferentially controlled by dynorphins through inhibition of GABA release from D1-MSN collaterals (**a**, **c**). The DYN-induced inhibition at this site would lead to disinhibition of D2-neurons. In contrast, in alcoholics with low DYN/KOR activity, the disinhibition of GABA release from D2-presynaptic terminals may lead to inhibition of D1-MSNs and, consequently, disinhibition of D2-MSNs (**b**, **d**)

Here, we tested this microcircuitry-based model and found a downregulation of *DRD1* expression in alcoholics. Importantly, the expression levels of the opioid *PDYN* and *OPRK1* genes and the *DRD1* and *DRD2* genes strongly correlate suggesting coordinated transcription of these genes, which apparently form a regulatory network that may control the function of the D1-/D2-MSN microcircuitry. Downregulation of *DRD1* expression supports the notion on low activity or a low number in D1-MSNs in NAc of alcoholics that may result in persistent disinhibition of the limbic system-evoked spiking of D2-MSNs (Fig. 5). This mechanism may contribute to D2-MSN activation underlying induction of aversive behavior and dysphoria. In conclusion, the microciruitry-based model obtained in preclinical experiments translates to the human brain.

A critical role for glial cells in substance addiction is becoming increasingly apparent (reviewed in [70, 71]). Opioids may directly activate glial cells in a non-classic opioid receptor manner, through the innate immune system’s pattern recognition receptor, toll-like receptor (TLR) 4. The opioid-induced glial activation apparently contributes to rewarding properties of these drugs. Most of the TLRs, particularly TLR4, are expressed in astroglia and microglia, the immune cells in the CNS. Glial inhibitors such as minocycline and selective TLR4 antagonism markedly reduce opioid-induced dependence and reward. Cellular and molecular mechanisms by which ethanol activates different signaling pathways also involve TLR4 and NLRP3, another neuroimmune target (reviewed in [70, 71]). Activation of these receptors results in the induction of cytokines and chemokines, which promote neuroinflammation, brain damage, behavioral and cognitive dysfunction, and addiction. We may speculate that the elevation in proportion of glial cells may be a factor that leads to dysregulation of glutamatergic and dopaminergic transmission in NAc of human alcoholics thus contributing to the formation of addicted state.

Our study focuses on changes in expression and co-expression levels of opioid and dopamine genes in alcoholics. Statistical analysis did not reveal effects of cell composition suggesting that the observed expression differences may be caused primarily by adaptations in transcriptional mechanisms. The algorithm of prediction of neuronal proportions from methylation levels has been validated using multiple metrics and applied in multiple studies [72–74]. A significant positive correlation between *RBFOX3* encoding neuronal nuclear antigen NeuN and neuronal proportion was evident in NAc (*P* = 4 × 10^−5^) [4]. These transcriptional changes may have functional consequences contributing to the dysregulation of the D1-/D2-MSN circuits in addicted brain. Importantly, observed downregulation of *DRD1* expression but unaltered *KOR* and *DRD2* mRNA in alcoholics corroborates our previously demonstrated decline in D1-receptor as well as unchanged D2- and KOR receptor-binding potential in NAc of human alcoholics and in rat model of alcohol dependence [15, 75]. Detailed information on the concentration of dynorphins in the vicinity of the receptor molecules is missing; at the present, it is challenging and virtually impossible to obtain such information for the human brain. What is known is that *PDYN* mRNA and PDYN-derived peptides correlate positively and with high significance in the human striatum (Pearson *R* = 0.63–0.73, *P* < 0.001) [35], supporting the notion on the usefulness of mRNA data for functional implementations.

In the following, we propose a role of transcriptional dysregulation of the D1-/D2-MSN microcircuitry within NAc in alcoholics even in the absence of complete information on biochemical properties and functional status of the DYN/KOR and D1- and D2-dopamine receptor systems in alcoholics. Two DYN/KOR mechanisms may differentially control processing of information in the MSN local circuitry (see Fig. 8 in [23]). The first mechanism may regulate GABA release from synaptic projections onto D1-neurons while the second one from those onto D2-neurons. Balance between these mechanisms may determine whether either D1- or D2-neurons are activated. The second mechanism requires high DYN/KOR activity, manifested as high levels of *PDYN* and *OPRK1* expression, and may be one of the factors underling persistent activation of D2-MSNs in a subgroup of alcoholics with enhanced DYN/KOR expression (Fig. 5a, c). Hypothetically, D2-MSNs could be also disinhibited when the MSN circuit is controlled by DYN at GABA-ergic synapses onto D1-neurons (Fig. 5b, d). At low activity of DYN/KOR system, GABA release may be enhanced leading to depression of D1-MSNs and consequently disinhibition of D2-MSNs. This low DYN/KOR activity may be based on lower *PDYN* and KOR expression levels in a subgroup of alcoholics.

Dopamine transmission from the ventral tegmental area (VTA) in NAc is critical for controlling both rewarding and aversive behaviors. Aversive behavior induced by silencing of (VTA) dopamine neurons is mediated by dopamine D2 receptors [21]. KOR is neuroanatomically positioned on the VTA DA terminals in NAc that enables dynorphins to inhibit DA release. Substantial evidence supports the concept of chronic alcohol-induced attenuation of DA [76, 77]. Stimulation of KOR produces dysphoria in humans [11] and place aversions in animals [10]. Increased DYN transmission in NAc induces depressive-like behavioral states in animal models of depression and negative affect [78–80]. Hypothetically, in alcoholics with high levels of PDYN and KOR expression, the increased DYN tone may result in attenuated dopaminergic transmission, produce depressive-like behaviors and dysphoria, and contribute to the increased alcohol consumption. Experimental studies support this hypothesis by demonstrating that KOR antagonist selectively reduces escalated operant self-administration of alcohol in dependent rats while leaving non-dependent alcohol self-administration intact [8, 26, 27]. Selective effects of KOR antagonists in dependent animals strongly implicate the recruitment of DYN/KOR system during transition to alcohol dependence [81].

On the other hand, both hypo- and hyperdopaminergia have been proposed as the states of vulnerability to relapse [15]. Dynamical changes may take place in the mesolimbic DA system during withdrawal and protracted abstinence resulting in hypodopaminergic state that characterizes acute withdrawal and a hyperdopaminergic state that characterizes protracted abstinence. While the hypodopaminergic state may be developed in human alcoholics characterized by elevated expression of PDYN and KOR, the low activity of this system in the subgroup of alcoholics may contribute to the development of the hyperdopaminergic state due to the reduced control of DA release from VTA projections. The hypothetical models fit to the existing addiction paradigms; however, they are speculative and require experimental validation in animal models and clinical settings.

The limitations of the study are first, that the findings are applicable to only males because no female subjects were analyzed. The second limitation is the absence of validation at the protein level of the main mRNA finding that is the interaction in expression of PDYN and KOR. There are no sufficiently sensitive biochemical and immunohistochemical methods including variants of western blotting [82] and high-resolution mass-spectrometry for quantitative analysis of the full-length PDYN and KOR proteins, which are expressed at very low levels. Immunohistochemical analysis of opioid receptors has not yet been well developed (for the controversies see [83]) while immunohistochemical analysis of PDYN is limited to the identification of antigenic epitopes of PDYN fragments but not the full-length molecule that is necessary for validation of the correlation. Furthermore, functional implementations of differences at the mRNA levels may be considered as “artificial.” However, having two alternatives, which are either (i) to avoid the functional interpretation of the findings or (ii) to discuss the data in frames of the established neurobiological models, we decided to follow the second option. The interpretation may not be straightforward; however, it may be useful for the development of molecular hypotheses and for the understanding of addiction processes especially in the human brain. Finally, the post-mortem human findings may be interpreted in two ways, either as molecular adaptations in DYN/KOR and *DRD1* expression patterns caused by chronic and excessive alcohol consumption and withdrawal or as manifestation of inherited molecular differences between controls and alcoholics.

In summary, our findings provide translational support of the notion that transcriptional dysregulation of DYN/KOR and dopamine signaling through both alteration in co-expression patterns of opioid genes and decreased *DRD1* gene expression may contribute to aberrant activity of the MSN microcircuitry within NAc which thereby may contribute to the negative affective state seen in human alcoholics. An understanding of the regulatory profiles of DYN/KOR system affected by chronic alcohol abuse may suggest alternative strategies for treating alcohol addiction. In a more general term, this study shows that co-expression analysis of gene clusters using post-mortem brain tissue can give new insights into the pathomolecular mechanism of a brain disease.

## Electronic supplementary material

All data generated or analyzed during this study are included in this published article.

Table S1 (DOCX 28 kb)

## Abbreviations

BAC: blood alcohol concentration
CI: confidence interval
CRH: corticotropin-releasing hormone
DA: dopamine
DR: dopamine receptor
*DRD1*: D1 dopamine receptor
*DRD2*: D2 dopamine receptor
DYN: dynorphin
GABA: gamma-aminobutyric acid
KOR: κ-opioid receptor
MSN: medium spiny neuron
NAc: nucleus accumbens
*OPRK1*: opioid receptor, kappa 1
*PDYN*: prodynorphin
PMI: post-mortem interval
*POLR2A*: RNA polymerase II subunit A
RQI: RNA quality indicator
*RPLP0*: ribosomal protein, large, P0
TLR: toll-like receptor
VTA: ventral tegmental area
