# Supplementary material for: Dynorphin and κ-Opioid Receptor Dysregulation in the Dopaminergic Reward System of Human Alcoholics
Source: Mol Neurobiol. 2018 Jan 30;55(8):7049–61. doi: 10.1007/s12035-017-0844-4 (PMC6061161; doi:10.1007/s12035-017-0844-4)
Supplement: Supplementary file 1 — (DOCX 28 kb) [file 12035_2017_844_MOESM1_ESM.docx]

**Supplemental Table 1** Demographic data and tissue characteristics of human subjects

| **Subject Id** | **Age** | **PMI** | **brain** | **RQI** | **BAC, g/100 mL** | **Smoking status** |
| --- | --- | --- | --- | --- | --- | --- |
|  | **(years)** | **(hours)** | **pH** |  |  |  |
| **Controls** |  |  |  |  |  |  |
| 1 | 48 | 17 | 6.71 | 7.9 | ? | No |
| 2 | 46 | 25 | 6.65 | 7.8 | ? | ? |
| 3 | 58 | 12 | 6.46 | 7.8 | ? | No |
| 4 | 50 | 19 | 6.26 | 8.0 | ? | Ex |
| 5 | 48 | 24 | 6.73 | 8.2 | ? | No |
| 6 | 43 | 66 | 6.2 | 7.1 | ? | No |
| 7 | 57 | 18 | 6.6 | 8.1 | ? | Ex |
| 8 | 73 | 48 | 6.8 | 8.2 | ? | Yes |
| 9 | 64 | 9.5 | 6.94 | 9.0 | ? | Yes |
| 10 | 53 | 27 | 6.64 | 7.4 | ? | ? |
| 11 * | 55 | 39 | 6.89 | 8.2 | ? | No |
| 12 * | 62 | 37.5 | 6.56 | 7.9 | ? | ? |
| 13 | 47 | 38 | 6.74 | 8.8 | 0.029 | Yes |
| 14 | 68 | 45.5 | 6.12 | 5.9 | – | No |
| 15 | 50 | 30 | 6.37 | 8.3 | – | Yes |
| 16 | 59 | 40 | 6.53 | 6.8 | – | Ex |
| 17 * | 56 | 19 | 6.9 | 8.3 | – | No |
| 18 | 55 | 12 | 6.39 | 8.9 | ? | No |
| 19 | 73 | 38.5 | 6.28 | 8.0 | ? | Ex |
| 20 * | 62 | 46 | 6.95 | 8.1 | – | Yes |
| 21 | 36 | 34 | 6.67 | 8.3 | – | Yes |
| 22 | 50 | 40 | 6.87 | 8.3 | – | Yes |
| 23 | 54 | 28 | 6.38 | 8.7 | ? | Yes |
| 24 | 58 | 28 | 5.92 | 3.3 | – | Yes |
| 25 | 69 | 52 | 6.95 | 8.5 | ? | No |
| 26 | 53 | 16 | 6.5 | 8.8 | – | Yes |
| 27 | 37 | 14.5 | 6.46 | 8.5 | – | No |
| 28 | 57 | 18 | 6.39 | 8.5 | – | Yes |
| 29 * | 50 | 34 | 6.77 | 8.7 | – | No |
| 30 | 59 | 29 | 6.61 | 8.8 | ? | No |
| 31 * | 37 | 24 | 6.7 | 7.7 | – | No |
| 32 * | 51 | 35 | 7.0 | 8.4 | – | Yes |
| 33 | 59 | 15 | 6.54 | 8.4 | – | No |
| 34 | 47 | 27 | 6.66 | 8.7 | – | Yes |
| 35 * | 64 | 29 | 6.55 | 7.7 | – | No |
| 36 | 61 | 22 | 6.41 | 8.8 | – | Yes |
| 37 | 40 | 27 | 6.79 | 8.9 | – | No |
| 38 | 61 | 30 | 6.69 | 8.8 | – | No |
| 39 * | 59 | 28.5 | 6.81 | 8.7 | ? | Yes |
| 40 | 40 | 59 | 6.93 | 8.9 | – | Ex |
| 41 | 64 | 30 | 6.82 | 8.1 | – | Yes |
| 42 * | 48 | 50 | 6.68 | 8.6 | 0.018 | No |
| 43 * | 46 | 26.5 | 6.68 | 8.7 | 0.007 | No |
| 44 * | 49 | 22 | 6.88 | 9.0 | – | Yes |
| 45 * | 59 | 49 | 6.86 | 8.1 | ? | No |
| 46 * | 55 | 23.5 | 6.88 | 9.0 | – | No |
| 47 * | 39 | 22 | 6.49 | 8.6 | – | Yes |
| 48 * | 50 | 29 | 6.82 | 8.4 | – | No |
| 49 * | 57 | 37 | 6.49 | 8.3 | 0.012 | No |
| 50 * | 55 | 25 | 6.08 | 7.1 | ? | Yes |
| **Alcoholics** |  |  |  |  |  |  |
| 1 | 54 | 17 | 6.41 | 7.7 | 0.016 | Yes |
| 2 | 50 | 24 | 6.59 | 7.3 | 0.241 | Yes |
| 3 * | 52 | 35 | 6.04 | 6.9 | 0.365 | ? |
| 4 | 70 | 19 | 6.34 | 5.7 | – | Yes |
| 5 | 50 | 17 | 6.3 | 6.3 | – | ? |
| 6 * | 51 | 46 | 6.35 | 6.4 | ? | Yes |
| 7 | 67 | 48 | 6.4 | 7.9 | – | Yes |
| 8 | 70 | 62 | 6.82 | 7.7 | – | Yes |
| 9 | 58 | 20 | 6.64 | 7.9 | – | Yes |
| 10 | 43 | 29 | 6.29 | 6.7 | ? | Ex |
| 11 | 73 | 43.5 | 6.59 | 7.1 | ? | No |
| 12 | 63 | 25.5 | 6.21 | 3.5 | – | Yes |
| 13 * | 40 | 40 | 6.42 | 8.3 | 0.2333 | Yes |
| 14 | 65 | 14.5 | 6.79 | 9.1 | ? | Ex |
| 15 | 55 | 48 | 7.02 | 7.5 | 0.246 | Yes |
| 16 | 64 | 39 | 6.76 | 8.5 | 0.293 | Yes |
| 17 | 55 | 17 | 6.85 | 9.0 | 0.206 | No |
| 18 | 59 | 35 | 6.57 | 5.6 | 0.063 | Yes |
| 19 | 61 | 27.5 | 5.87 | 5.6 | – | Yes |
| 20 | 58 | 44.5 | 6.47 | 8.1 | – | Yes |
| 21 | 65 | 72 | 6.88 | 7.1 | – | Yes |
| 22 | 69 | 22 | 5.82 | 5.7 | – | Yes |
| 23 | 43 | 33 | 6.57 | 8.4 | 0.103 | Yes |
| 24 | 61 | 59 | 6.57 | 8.1 | – | Yes |
| 25 | 49 | 44 | 6.41 | 8.5 | 0.03 | Yes |
| 26 * | 62 | 40 | 6.59 | 7.5 | 0.24 | Yes |
| 27 | 63 | 28 | 6.89 | 9.1 | – | Yes |
| 28 | 62 | 30.5 | 6.79 | 9.0 | – | Yes |
| 29 * | 44 | 59 | 6.87 | 8.2 | 0.309 | Yes |
| 30 * | 55 | 27.5 | 6.56 | 6.7 | – | Yes |
| 31 * | 60 | 28 | 6.48 | 6.5 | ? | Yes |
| 32 * | 50 | 34.5 | 6.93 | 8.5 | 0.395 | No |
| 33 * | 61 | 52 | 6.63 | 7.3 | – | Yes |
| 34 * | 47 | 72 | 5.92 | 3.8 | 0.132 | No |
| 35 * | 40 | 50.5 | 6.83 | 8.4 | – | No |
| 36 * | 54 | 22.5 | 6.77 | 8.6 | 0.38 | No |
| 37 * | 51 | 51.5 | 6.8 | 7.7 | 0.02 | Yes |
| 38 * | 53 | 53 | 6.77 | 7.6 | 0.165 | Yes |
| 39 * | 52 | 47.5 | 6.7 | 7.5 | 0.430 | Yes |
| 40 * | 47 | 36 | 6.51 | 8.6 | 0.037 | Yes |
| 41 * | 55 | 24 | 6.43 | 8.1 | 0.292 | Yes |
| 42 * | 56 | 39.5 | 6.34 | 8.2 | – | Yes |

Subject Id, subject’s depersonalized identification number; PMI, postmortem interval; RQI, RNA quality indicator; BAC, blood alcohol concentration.

* neuronal proportions were not measured
